# Factors associated with neonatal sepsis among neonates admitted in Kibungo Referral Hospital, Rwanda

**DOI:** 10.1038/s41598-024-66818-z

**Published:** 2024-07-10

**Authors:** Jean Claude Niyoyita, Jerome Ndayisenga, Jared Omolo, Hosee Niyompano, Pierre Celestin Bimenyimana, Tafadzwa Dzinamarira, Olivier Nsekuye, Isabella Chavez, François Hakizayezu

**Affiliations:** 1 Rwanda Field Epidemiology and Laboratory Training Program, Department of Biostatistics and Epidemiology, School of Public Health, University of Rwanda, P.O. Box 3286, Kigali, Rwanda; 2 African Research and Community Health Initiative, Kigali, Rwanda; 3 University of Pretoria, Pretoria, South Africa; 4 Rwanda Biomedical Centre, Kigali, Rwanda; 5 University of California, Los Angeles (UCLA), Los Angeles, USA

**Keywords:** Neonatal sepsis, Neonates, Risk factors, Rwanda, Diseases, Medical research

## Abstract

More than one million neonatal deaths occur every year worldwide, of which 99% take place in low-income countries. In Rwanda, nearly 71% of neonatal deaths are preventable and among these, 10% are due to neonatal sepsis. Nevertheless, limited information exists on neonatal sepsis and its associated factors in Rwanda. The objectives of the study were to find prevalence and factors associated with neonatal sepsis among neonates admitted in Kibungo Referral Hospital, Ngoma District, Rwanda. We used a retrospective cross-sectional study design reviewing a subset of neonatal, maternal and laboratory records from Kibungo Hospital in 2017. Data were reviewed and collected from March to May, 2018. Logistic regression and odds ratios were calculated to identify the factors associated with neonatal sepsis at 95% CI, *p* < 0.05. Of the 972 total neonates’ medical records from 2017, we randomly selected 422 of which 12.8% (n = 54) had neonatal sepsis. When blood cultures were positive, 62% grew Klebsiella *pneumoniae*. Among neonates with sepsis, 38 (70%) recovered while 16 (30%) died. Neonatal sepsis was strongly associated with neonatal age less than or equal to three days (aOR: 2.769, 95% CI 1.312–5.843; *p* = 0.008); and gestational age less than 37 weeks (aOR: 4.149; CI 1.1878–9.167; *p* ≤ 0.001). Increased use of blood cultures including sensitivity testing, routine surface cultures of the neonatology and maternity wards facilities, and systematic ward cleaning are all important approaches to prevent and treat neonatal infections in additional to regular neonatal sepsis evaluations.

## Introduction

Neonatal sepsis remains a public health concern worldwide. More than one million neonatal deaths occur per year, with 99% occurring in low- and middle-income countries^1–3^. The global neonatal mortality rate has decreased over the past years, but to a lesser extent compared to other under-five child deaths^4^. Globally, an estimate of 22, 25 and 34% of under-five deaths resulted from neonatal infections, intrapartum related conditions and preterm birth complications respectively, with highest mortality rates being reported in Sub-Saharan Africa^5^. Neonatal deaths account for 68% of under-five deaths in Rwanda^6^. Rural hospitals mainly serve the poor community, and have limited resources for controlling infectious diseases which might increase the risks of developing sepsis. However, proper management of neonatal sepsis can improve patient outcomes^7–12^.

Neonatal sepsis is defined as bloodstream infection resulting from bacterial, viral and or fungal pathogens occurring within 28 days after birth^13^. It is subcategorized as early and late onset sepsis, occurring before and after 72 h of life, respectively^14–17^. Early-onset sepsis is typically due to the pathogens acquired during peripartum period, prior to or during childbirth^18^. Late onset sepsis is generally due to hospital acquired infections after birth^19–22^, and unenforced infection prevention and control in the Hospital settings^23–27^. The 72-h cut-off point was selected for this study due to the wide range review of most recent articles^28–30^. Laboratory evaluations of neonatal sepsis include an elevated or depressed white blood cell count with a left shifted differential count, an elevated C-Reactive Protein, and a positive blood culture^31,32^. However, the blood culture which is the gold standard in sepsis confirmation, is not routinely available in low-income countries^1,33^.

Neonatal sepsis prevalence is between 1 and 10 per 1,000 live births in developed countries but increases threefold in low-income countries. The prevalence of early and late onset neonatal sepsis varies by region. For example, the incidence of neonatal mortality was higher in rural than in urban settings in Senegal and in Kenya^34,35^. Factors reported to be associated with sepsis also vary by region and include young maternal age, poor access to antenatal care, post-natal age of neonate, low birth weight, and birth asphyxia^36–41^. The neonatal mortality rate is high in Rwanda at 19 out of 1,000 live births according to the 2019–2020 Rwanda Demographic Health Survey (RDHS)^36^. About 71% of neonatal deaths in Rwanda are preventable and neonatal sepsis accounts for 10% of total preventable causes; and along with sepsis, asphyxia and complications of prematurity are among the leading causes of neonatal deaths in Rwanda^42^.

Only a few studies on neonatal sepsis have been conducted in Rwanda, and most of them focused on the urban setting^43–45^. Kibungo is a rural referral hospital located approximately 100 km from Kigali. It is the main referral facility for 12 health centers in its catchment area with only one neonatology unit serving about 85 neonates monthly. To our knowledge, paucity of studies has been conducted to assess risk factors associated with neonatal sepsis among neonates admitted in Ngoma District. This study aimed to determine the prevalence and factors associated with neonatal sepsis among neonates admitted in Kibungo Referral Hospital, Ngoma District of Rwanda.

## Methods

### Study design and setting

This retrospective, cross-sectional study reviewed the medical records of all neonates admitted at Kibungo Hospital, Ngoma District, Eastern Province, Rwanda from January-December 2017. Kibungo Hospital has a 312 bed capacity including 24 beds in the Neonatology ward. Kibungo Hospital Neonatology services include Neonatology Intensive Care (Unit) and Kangaroo mother care. Patients are cared for by a multidisciplinary team comprised by Pediatricians, General practitioners(GPs) and Nurses.

### Study population and sample size

Of the 972 neonatal medical records at Kibungo Hospital from 2017, we collected neonatal and maternal data from a random sample of 422. We also collected all environmental laboratory cultures sent from the neonatology and maternity wards during 2017.

### Data collection and procedures

Clinical data including neonatal and maternal characteristics were extracted from neonatal files. Environment swabbing culture results were collected from laboratory records. A Microsoft Excel data collection tool was developed to record neonatal, maternal and ward environmental data. Data collection was conducted in a three-month period from March to May, 2018.

Maternal data were: age, fever > 38 °C, number of previous pregnancies, place of delivery, educational level, employment status, health insurance, obstructed labor, premature rupture of membrane (PROM), foul smelling amniotic fluid. Neonatal data were: post-natal age, sex; birth weight; mode of delivery; place of birth; birth asphyxia (yes/no); gestational age in weeks; Apgar score at 10 min; resuscitation after birth (yes/no); sepsis (yes/no), and if yes, time of onset, bacterial isolate, and sepsis outcome (died or recovered). The determination of sepsis was relied on physicians’ classification of whether the neonate had sepsis as final diagnosis.

The environment cultures data were gathered from laboratory records. Laboratory records were reviewed to collect information related to environmental samples collected for bacteriological cultures, sample sources and identified bacteria species.

### Data analysis

Stata Statistical Software was used for analysis: version 13. College Station, TX: Stata Corp LP https://www.stata.com/. Descriptive statistics, including frequencies and percentages, were calculated to characterize the study population in terms of socio-demographic and other relevant variables. Analytical statistics were performed for bivariate and multivariable logistic regression. For the bivariate logistic regression, the association between independent and dependent variables was found at 95% confidence interval with *p* value < 0.05. Multivariable logistic regression was done for the variables that showed significant association at the bivariate analysis, and *p* value ≤ 0.05 was considered statistically significant.

### Ethical approval

The study obtained ethical approval from the ethical committee of Kibungo Referral Hospital with a reference letter (Ref No:14/117/RJ/H1-1/2/2018). And all data were collected in accordance with all relevant guidelines and in the Declaration of Helsinki^46^. The secondary data from a programmatic management health system were used hence the informed consent and assent were waived by the ethical committee of Kibungo Referral Hospital. Findings of the study were submitted to Kibungo Hospital and the University of Rwanda, College of Medicine and Health Sciences, School of Public Health.

## Results

### Neonatal characteristics

A total of 422 neonates admitted to the neonatology department in 2017 were randomly selected, and their records reviewed. Among the 54 with neonatal sepsis 57% (31/54) were early onset and 43% (23/54) were late onset sepsis, and 38 (70%) recovered, while 16 (30%) died (Table 1).

**Table 1:** Socio-demographic and clinical characteristics of neonates admitted at Kibungo Hospital neonatology unit, January–December, 2017 (n = 422).

Variables	Frequencies (n)	Percentages (%)
Sex		
Male	216	51.18
Female	206	48.82
Age group in days		
0–3	369	87.44
4–28	53	12.56
Birth weight		
< 2500	213	50.47
≥ 2500	209	49.53
Mode of delivery		
Caesarean	94	22.27
Vaginal	328	77.73
Place of birth		
Hospital	251	59.48
Health center	135	31.99
Home	36	8.53
Birth asphyxia		
Yes	85	56.64
No	337	43.36
Gestational Age (Weeks)		
< 37	239	56.6
≥ 37	183	43.4
Apgar at 10 min n = 357		
≤ 6	85	23.81
> 6	272	76.19
Resuscitated		
Yes	98	23.20
No	324	76.80
Sepsis		
Yes	54	12.80
No	368	87.20
Sepsis category n = 54		
Early onset	31	57
Late onset	23	43
Sepsis outcome n = 54		
Recovered	38	70
Died	16	30
Bacterial isolate		
*Acinetobacter baumanii*	1	4.76
*Coagulase neg staphylococci*	2	9.52
*Escherichia* Coli	2	9.52
*Klebsiella* pneumonia	13	61.90
*Staphylococcus aureus*	1	4.76
*Streptococcus pneumonia*	2	9.52

### Neonates’ mothers’ characteristics

Almost seven percent (7%) of the mothers experienced more than five pregnancies; most of them were employed or self-employed; most of the participants had health insurance (Table 2).

**Table 2:** Socio-demographic characteristics of neonates’ mothers at Kibungo Hospital neonatology unit, January to December, 2017 (N = 422).

Variables	Frequencies (n)	Percentages (%)
Obstructed labor		
Yes	7	1.7
No	415	98.3
Maternal fever		
Yes	40	9.5
No	382	90.5
Number of previous pregnancies		
< 5	394	93.36
5 and above	28	6.64
Meconium stained amniotic fluid		
Yes	53	12.56
No	369	87.44
Maternal infection		
Yes	3	0.71
No	419	99.29
Premature Rupture of Membrane (PROM)		
Yes	32	7.58
No	390	92.42
Foul smelling		
Yes	8	1.89
No	414	98.11
Age group in years		
17–20	59	13.9
21–30	254	60.2
> 30	109	25.9
Employment status		
Employed	416	98.6
Unemployed	6	1.4
Education level		
None	9	2.1
Primary	371	87.9
Secondary and high	42	10.0
Health insurance		
Yes	396	93.8
No	26	6.2

### Neonatal sepsis characteristics, antibiotic susceptibility pattern and outcome

Fifty-four (12.8%) neonates were diagnosed with sepsis of whom 25 (46.3%) had blood cultures performed. Almost 84% (21/25) of the blood cultures were positive with *Klebsiella pneumoniae* predominance (62%), followed by coagulase-negative staphylococci (9.5%), *Escherichia coli* (9.5%), *Streptococcus pneumoniae* (9.5%), *Acinetobacter baumanii* (4.7%) and *Staphylococcus aureus* (4.7%). Bacteria sensitivity pattern revealed that 46.1% of the *Klebsiella pneumoniae* was resistant to ciprofloxacin but 100% were sensitive to meropenem and imipenem in carbapenems family of medications. Only 70.4% of neonates with sepsis survived (Table 1).

### Environmental bacterial colonization monitoring

Environmental cultures reviewed were collected from surfaces of neonatology and maternity equipment such as incubators, lamps, nasal aspirator and oxygen concentrators, and showed the presence of various bacterial species in the neonatal and maternity wards: *Enterobacter cloacae*, *Klebsiella pneumonia*e, *Serratia* species, *Providencia stuartii* and *Escherichia coli*.

### Bivariate analysis of neonatal characteristics

After bivariate analysis, neonates with a younger post-natal age group (*p* = 0.022) and younger gestational age (*p* = 0.031) were found to be significantly associated with neonatal sepsis (Table 3) and therefore, they were considered for multivariate logistic regression analysis. Association of Apgar score with neonatal sepsis was not statistically significant (*p* = 0.771).

**Table 3:** Bivariate and multivariate analysis of neonatal characteristics associated with sepsis at Kibungo Hospital.

Variables	N = 422	Sepsis developed				cOR (95% CI)	*p* value	aOR (95% CI)	*p* value
		Yes		No					
		No	%	No	%				
Sex									
Male	216	26	12.04	190	87.96	1	0.633		
Female	206	28	13.59	178	86.41	0.869 (0.491–1.540)			
Age group (days)									
≤ 3	369	42	11.38	327	88.62	**2.278 (1.110–4.677)**	**0.022**	**2.769(1312–5.8430**	0.008*
≥ 4	53	12	22.64	41	77.36	1		1	
Birth weight									
< 2500	213	32	15.02	181	84.98	1	0.167		
≥ 2500	209	22	10.53	187	89.47	1.502 (0.841–2.684)			
Mode of delivery									
Caesarean	94	8	8.51	86	91.49	1	0.163		
Vaginal	328	46	14.02	282	85.98	0.570 (0.259–1.254)			
Birth asphyxia									
Yes	85	11	12.94	74	87.06	1	0.964		
No	337	43	12.76	294	87.24	1.016 (0.499–2.066)			
Gestational age (weeks)									
< 37	239	38	15.90	201	84.10	**2.573 (1.358–4.875)**	**0.004**	**4.149 (1.878–9.167)**	
≥ 37	183	16	8.74	167	91.26	1		1	< 0.001**
Apgar score n (357)									
≤ 6	85	11	12.94	74	87.06	1	0.771		
≥ 7	272	32	11.76	240	88.24	1.114 (0.535–2.320)			

*: Statistical significance at *p* < 0.05, **: statistical significant at *p* < 0.01.

*cOR* crude odds ratio, *aOR* adjusted odds ratio.

Significant values are in [bold].

### Bivariate analysis of maternal characteristics

After the bivariate analysis of the maternal characteristics, it was seen that no maternal characteristic was statistically significant and eligible for the multivariable logistic regression (Table 4).

**Table 4:** Bivariate analysis of maternal characteristics associated with sepsis at Kibungo Hospital.

Variables	Sepsis developed					cOR (95% CI)	*p* value
	N = 422	Yes		No			
		Frequency	%	Frequency	%		
Age group							
17–20	59	5	8.47	54	91.53	1.726 (0.645–4.613)	0.277
21–30	254	35	13.78	219	86.22	1	
> 30	109	14	12.84	95	87.16	1.084 (0.557–2.108)	0.811
Education level							
No education	9	2	22.22	7	77.78	1	
Primary	371	45	12.13	324	87.87	2.069 (0.417–10.273)	0.373
Secondary and higher	42	7	16.67	34	83.33	1.42 (0.243–8.375)	0.693
Occupation							
Employed	416	54	12.98	362	87.02	1	
Unemployed	6	0	0	6	100.00	1(Omitted)	0.345
Place of delivery							
Hospital	251	28	11.16	223	88.84	1	
Health center	135	20	14.81	115	85.19	0.721 (0.389–1.337)	0.300
Home	36	6	16.67	30	83.33	0.627 (0.240–1.640)	0.342
Premature Rupture of Membrane (PROM)							
Yes	32	4	12.50	28	87.50	1	
No	390	50	12.82	340	87.18	0.971 (0.326–2.886)	0.958
Foul smelling							
Yes	8	2	25.00	6	75.00	1	
No	414	52	12.56	362	87.44	2.320 (0.456–11.802)	0.310
Obstructed labor							
Yes	7	1	14.29	6	85.71	1	
No	415	53	12.77	362	87.23	1.138 (0.134–9.642)	0.905
Maternal fever							
Yes	40	7	17.50	33	82.50	1	
No	382	47	12.30	335	87.70	0.413 (0.457–1.284)	0.352
Health insurance							
Yes	396	51	12.88	345	87.12	1	
No	26	3	11.54	23	88.46	1.133 (0.328–3.910)	0.843

### Multivariable analysis of characteristics associated with sepsis

Multivariable analysis was done only for the neonate characteristics that showed significant association with neonatal sepsis in bivariate analysis (neonate age and gestational weeks). Strong association with sepsis was found with neonatal age less or equal to three days (aOR: 2.769; 95% CI 1.312–5.843; *p* = 0.008), and gestational weeks less than 37 weeks (aOR: 4.149; CI 1.1878–9.167; *p*  ≤  0.001) (Table 3).

## Discussion

The purpose of this study was to identify the risk factors associated with neonatal sepsis among neonates admitted in Kibungo Referral Hospital, Ngoma District, Rwanda during the calendar year of 2017. Neonatal sepsis prevalence was 12.8%, of whom 29.6% died. Decreasing deaths for newborns and mothers is a global priority to achieve Sustainable Development Goals, and to implement United Nations Global Strategy for Women, Children and Adolescent health^5^. Though Rwanda is among the few countries in Africa that achieved the fourth Millennium Development Goal to reduce child mortality, mortality rate among neonates is still of great concern. Neonatal sepsis is still a leading cause of neonatal morbidity and mortality in Rwanda, including in the study area^47,48^.

The 12.8% neonatal sepsis prevalence found in this study is almost similar to results reported in the Northwest Ethiopia (11.7%)^49^. But it is lower compared to reports from Ethiopia (33.8%), Tanzania (24%)^50^. The difference in neonatal sepsis prevalence may be due to the difference in the definitions of sepsis and the study settings in terms of infection prevention and control, staffing, funding, and policy.

In this study, early onset neonatal sepsis was slightly more common (57.4%) than late onset neonatal sepsis (42.6%). On the other hand, our study results contrasts with what was reported in a research conducted at Mansoura Hospital in Egypt, where early onset sepsis was lower at 44.2% compare to late onset sepsis at 55.8%^51^. This could be due to the differences in the characteristics of mothers and the setting where the study was conducted.

The sepsis fatality rate in this study was 29.6%, which is high and may reflect inadequate management of sepsis, perhaps linked to delays in diagnosis and treatment. This sepsis fatality rate is similar to the findings of a study from Tehran (27.4%)^52^. Blood cultures were not routinely performed in suspected cases, with only 25 of the 54 cases of suspected sepsis confirmed by culture. This could be due to the long interval between sending and receiving the culture results that might push physicians to treat suspected sepsis empirically. The predominance of *Klebsiella pneumoniae* was noted and the sensitivity pattern was similar to the findings from the research conducted in India where the isolated bacteria were sensitive to Meropenem and Imipenem with almost half of the isolated *Klebsiella pneumonia* bacteria species were resistant to Ciprofloxacin^53^. The findings of current study are different from a study done in Sudan that reported susceptibility of *Klebsiella pneumoniae* of 87% to Ciprofloxacin, and 81% to meropenem, which was caused by a variation of antimicrobial susceptibility patterns in bacteria^54^.

Multivariable analysis revealed that younger gestational age and postnatal age were both statistically significantly associated with neonatal sepsis. Neonates aged 3 days or less were more likely to develop sepsis which is comparable with the study done in Ethiopia neonates^55,56^. Premature rupture of membrane, place of delivery, intrapartum fever, Apgar Score < 7 at 10 min, low birth weight, meconium stained amniotic fluid, foul smelling amniotic fluid and assisted ventilation were not found to be associated factors, in contrast to the studies done in Mexico and Ethiopia which, of note, were conducted in urban settings^19,57^.

In this study, environmental cultures showed the presence of *Enterobacter cloacae*, *Klebsiella pneumoniae*, *Serratia species*, *Providencia stuartii* and *Escherichia coli* in the neonatal and maternity wards. The study done in Morocco, Brazil, Austria and India on neonatology environment reported the presence of different bacteria including *Klebsiella pneumonia*e, *coagulase*-*negative staphylococci* and other *Enterobacteriaceae*^37,58–60^. The presence of those bacteria isolates in the neonatal and maternity settings highlights the need to regularly monitor their environment and execute infection control by improving hygiene and sanitation.

## Limitation of the study

One of the study limitation was the fact that data were collected in a single rural referral hospital in Rwanda. Therefore, the study results may not be directly applicable to other settings. Additionally, the use of secondary data may have limited the available data to assess for statistical association with neonatal sepsis in the study area.

## Conclusion

The study highlighted that post-natal age ≤ 3 days, and gestation age < 37 weeks were significantly associated with neonatal sepsis. Improving the use of blood culture and consistent tailoring of antibiotics based on antibiotics susceptibility testing could enhance the management of neonatal sepsis.

## Data Availability

The datasets used and/or analyzed during the current study is available from the corresponding author on reasonable request.

## References

[1] Pillay D, Naidoo L, Swe Swe-Han K, Mahabeer Y (2021). Neonatal sepsis in a tertiary unit in South Africa. BMC Infect. Dis..

[2] Oo NAT (2021). Neonatal sepsis, antibiotic susceptibility pattern, and treatment outcomes among neonates treated in two tertiary care hospitals of Yangon, Myanmar from 2017 to 2019. Trop. Med. Infect. Dis..

[3] Waters D (2011). Aetiology of community-acquired neonatal sepsis in low- and middle-income countries. J. Glob. Health.

[4] Uwingabire E, Tengera O, Batamuriza M, Mukamana D (2020). Umbilical cord care among postnatal mothers in Kibungo Hospital catchment area, Rwanda. Rwanda J. Med. Health Sci..

[5] Lawn, J. E. *et al.* Every newborn 2 progress, priorities, and potential beyond survival. Lancet **6736** (2015). 10.1016/S0140-6736(14)60496-7 24853593

[6] UNICEF. *2018-Situation-Analysis-Rwanda-Children-Full-Report*. *Situation Analysis of Children in Rwanda* (2018).

[7] Wale A, Chelkeba L, Wobie Y, Abebe A (2021). Treatment outcome and associated factors of neonatal sepsis at Mizan Tepi University teaching hospital, South West Ethiopia: A prospective observational study. Pediatr. Heal. Med. Ther..

[8] Niederman MS (2021). Initial antimicrobial management of sepsis. Crit. Care.

[9] Byiringiro S (2021). A qualitative study to explore the experience of parents of newborns admitted to neonatal care unit in rural Rwanda. PLoS One.

[10] Hoque M, Haaq S, Islam R (2011). Causes of neonatal admissions and deaths at a rural hospital in KwaZulu-Natal, South Africa. S. Afr. J. Epidemiol. Infect..

[11] Viswanathan R (2012). Profile of neonatal septicaemia at a district-level sick newborn care unit. J. Heal. Popul. Nutr..

[12] McAdams RM (2015). Implementation of bubble CPAP in a rural Ugandan neonatal ICU. Respir. Care.

[13] Bulbul A (2020). Neonatal sepsis. Sisli Etfal Hastan. Tip Bul..

[14] Simonsen KA, Anderson-Berry AL, Delair SF, Dele Davies H (2014). Early-onset neonatal sepsis. Clin. Microbiol. Rev..

[15] Lebea MM, Davies V (2017). Evaluation of culture-proven neonatal sepsis at a tertiary care hospital in Johannesburg, South Africa. SAJCH S. Afr. J. Child Heal..

[16] Arowosegbe AO, Ojo DA, Dedeke IO, Shittu OB, Akingbade OA (2017). Neonatal sepsis in a Nigerian Tertiary Hospital: Clinical features, clinical outcome, aetiology and antibiotic susceptibility pattern. S. Afr. J. Infect. Dis..

[17] Mhada TV, Fredrick F, Matee MI, Massawe A (2012). Neonatal sepsis at Muhimbili National Hospital, Dar es Salaam, Tanzania; Aetiology, antimicrobial sensitivity pattern and clinical outcome. BMC Public Health.

[18] Silveira RDC (2019). The challenges of neonatal sepsis management. J. Pediatr..

[19] Gebremedhin D, Berhe H, Gebrekirstos K (2016). Risk factors for neonatal sepsis in public hospitals of Mekelle City, North Ethiopia, 2015: Unmatched case control study. PLoS One.

[20] World Health Organisation. *Global report on the epidemiology and burden of sepsis: Current evidence, identifying gaps and future directions* (World Health Organization, 2020).

[21] Seale AC, Mwaniki M, Newton CR, Berkley JA (2009). Maternal and early onset neonatal bacterial sepsis: Burden and strategies for prevention in sub-Saharan Africa. Lancet Infect. Dis..

[22] Veen AV (2011). Outbreak of infection with a multiresistant *Klebsiella pneumoniae* strain associated with contaminated roll boards in operating rooms. J. Clin. Microbiol..

[23] Seale AC (2013). Neonatal severe bacterial infection impairment estimates in South Asia, sub-Saharan Africa, and Latin America for 2010. Pediatr. Res..

[24] Huynh BT (2015). Burden of bacterial resistance among neonatal infections in low income countries: How convincing is the epidemiological evidence?. BMC Infect. Dis..

[25] Agarwal R (2016). Characterisation and antimicrobial resistance of sepsis pathogens in neonates born in tertiary care centres in Delhi, India: A cohort study. Lancet Glob. Heal..

[26] Alcock G, Liley HG, Cooke L, Gray PH (2017). Prevention of neonatal late-onset sepsis: A randomised controlled trial. BMC Pediatr..

[27] Alam MM, Saleem AF, Shaikh AS, Munir O, Qadir M (2014). Neonatal sepsis following prolonged rupture of membranes in a tertiary care hospital in Karachi, Pakistan. J. Infect. Dev. Ctries..

[28] Cortese F (2016). Early and late infections in newborns: Where do we stand? A review. Pediatr. Neonatol..

[29] Ogundare E (2019). Presentation and outcomes of early and late onset neonatal sepsis in a Nigerian hospital. Afr. Health Sci..

[30] Dong Y, Speer CP (2015). Late-onset neonatal sepsis: Recent developments. Arch. Dis. Child. Fetal Neonatal Ed..

[31] Popowski T (2011). Maternal markers for detecting early-onset neonatal infection and chorioamnionitis in cases of premature rupture of membranes at or after 34 weeks of gestation: A two-center prospective study. BMC Pregnancy Childbirth.

[32] Rwanda Ministry of Health. *National Neonatal Care Protocal* (2020).

[33] Mkony MF, Mizinduko MM, Massawe A, Matee M (2014). Management of neonatal sepsis at Muhimbili National Hospital in Dar es Salaam: Diagnostic accuracy of C—reactive protein and newborn scale of sepsis and antimicrobial resistance pattern of etiological bacteria. BMC Pediatr..

[34] Hutchinson Z, Cisse AM, Elewonibi B, BeLue R (2017). Neonatal outcomes in a community hospital in M’Bour, Senegal. J. Glob. Health.

[35] Ameyaw E, Asafo Agyei SB, Plange Rhule G (2017). Spectrum of Diseases seen on Neonatal Ward at Komfo Anokye Teaching Hospital, Kumasi, Ghana. Pediatr. Infect. Dis. Open Access.

[36] National Institute of Statistics of Rwanda (NISR) [Rwanda], Ministry of Health (MOH) [Rwanda], & ICF. Rwanda Demographic and Health Survey 2019–20 Final Report. Kigali, Rwanda, and Rockville, Maryland, USA: ICF, NISR (2021).

[37] John B, David M, Mathias L, Elizabeth N (2015). Risk factors and practices contributing to newborn sepsis in a rural district of Eastern Uganda, August 2013: a cross sectional study. BMC Res. Notes.

[38] Getabelew A, Aman M, Fantaye E, Yeheyis T (2018). Prevalence of neonatal sepsis and associated factors among neonates in neonatal intensive care unit at selected governmental hospitals in Shashemene Town, Oromia Regional State, Ethiopia, 2017. Int. J. Pediatr..

[39] Jabiri A, Wella HL, Semiono A, Sariah A, Protas J (2016). Prevalence and factors associated with neonatal sepsis among neonates in Temeke and Mwananyamala Hospitals in Dar es Salaam, Tanzania. Tanzan. J. Health Res..

[40] Medhat H, Khashana A, El-Kalioby M (2016). Incidence of neonatal infection in South Sinai, Egypt. Int. J. Infect..

[41] Afsharpaiman S (2012). Trends in incidence of neonatal sepsis and antibiotic susceptibility of causative agents in two neonatal intensive care units in Tehran, I.R Iran. J. Clin. Neonatol..

[42] Khurmi MS (2017). Newborn survival case study in Rwanda—Bottleneck analysis and projections in key maternal and child mortality rates using lives saved tool (LiST). Int. J. Matern. Child Health AIDS.

[43] Ndayizeye R, Sibomana E, Nyaziyose I, Conard CJ, Cartledge P (2019). Neonatal antibiotic use at a district and teaching hospital in Rwanda-a retrospective descriptive study. Rwanda Med. J..

[44] Nimukuze E, Mukarwego B, Bizimana GE, Rutayisire E (2021). Trends and factors associated with neonatal sepsis at Ruhengeri Referral Hospital, Rwanda. Austin J. Womens Health.

[45] Cartledge PT, Ruzibuka FS, Rutagarama F, Rutare S, Rogo T (2020). Antibiotic prescribing practices in three neonatology units in Kigali, Rwanda. An observational study. Afr. Health Sci..

[46] Shrestha B, Dunn L (2020). The declaration of Helsinki on medical research involving human subjects: A review of seventh revision. J. Nepal Health Res. Council.

[47] Lozano R (2012). Global and regional mortality from 235 causes of death for 20 age groups in 1990 and 2010: A systematic analysis for the Global Burden of Disease Study 2010. Lancet.

[48] Nyishime M (2018). A retrospective study of neonatal case management and outcomes in rural Rwanda post implementation of a national neonatal care package for sick and small infants. BMC Pediatr..

[49] Yismaw AE, Abebil TY, Biweta MA, Araya BM (2019). Proportion of neonatal sepsis and determinant factors among neonates admitted in University of Gondar comprehensive specialized hospital neonatal Intensive care unit Northwest Ethiopia 2017. BMC Res. Notes.

[50] Mersha A (2019). Neonatal sepsis and associated factors among newborns in hospitals of Wolaita Sodo Town, Southern Ethiopia. Res. Rep. Neonatol..

[51] Shehab El-Din EM, El-Sokkary MM, Bassiouny MR, Hassan R (2015). Epidemiology of neonatal sepsis and implicated pathogens: A study from Egypt. Biomed. Res. Int..

[52] Afsharpaiman S (2012). Trends in incidence of neonatal sepsis and antibiotic susceptibility of causative agents in two neonatal intensive care units in Tehran, I.R. Iran. J. Clin. Neonatol..

[53] Viswanathan R (2012). Multi-drug resistant gram negative bacilli causing early neonatal sepsis in India. Arch. Dis. Child. Fetal Neonatal Ed..

[54] Gasim Khalil EA (2019). Late onset neonatal sepsis in Sudan: Incidence, bacteriological profiles, patterns of antimicrobial resistance and fatality. Acad. J. Pediatr. Neonatol..

[55] Geyesus T, Moges F, Eshetie S, Yeshitela B, Abate E (2017). Bacterial etiologic agents causing neonatal sepsis and associated risk factors in Gondar, Northwest Ethiopia. BMC Pediatr..

[56] Schrag SJ (2012). Risk factors for neonatal sepsis and perinatal death among infants enrolled in the prevention of perinatal sepsis trial, Soweto. South Africa. Pediatr. Infect. Dis. J..

[57] Leal YA (2012). Risk factors and prognosis for neonatal sepsis in southeastern Mexico: Analysis of a four-year historic cohort follow-up. BMC Pregnancy Childbirth.

[58] Van’t Veen A (2005). Outbreak of infection with a multiresistant *Klebsiella pneumoniae* strain associated with contaminated roll boards in operating rooms. J. Clin. Microbiol..

[59] Tewabe T (2017). Clinical outcome and risk factors of neonatal sepsis among neonates in Felege Hiwot referral Hospital, Bahir Dar, Amhara Regional State, North West Ethiopia 2016: A retrospective chart review. BMC Res. Notes.

[60] Mathur S, Fuchs A, Bielicki J, Van Den Anker J, Sharland M (2018). Antibiotic use for community-acquired pneumonia in neonates and children: WHO evidence review. Paediatr. Int. Child Health.

